# Chikungunya virus perturbs the Wnt/β-catenin signaling pathway for efficient viral infection

**DOI:** 10.1128/jvi.01430-23

**Published:** 2023-10-20

**Authors:** Sanchari Chatterjee, Soumyajit Ghosh, Ankita Datey, Chandan Mahish, Subhasis Chattopadhyay, Soma Chattopadhyay

**Affiliations:** 1 Institute of Life Sciences, Bhubaneswar, India; 2 Regional Centre for Biotechnology, Faridabad, India; 3 School of Biotechnology, Kalinga Institute of Industrial Technology (KIIT) University, Bhubaneswar, India; 4 National Institute of Science Education and Research, an OCC of Homi Bhaba National Institute, Bhubaneswar, Odisha, India; Loyola University Chicago, Maywood, Illinois, USA

**Keywords:** Chikungunya virus, Wnt/β-catenin signaling pathway, iCRT14, CHIKV-nsP2, β-catenin

## Abstract

Viruses use various strategies to modulate host pathways and take benefit of host machineries for efficient infection. It has been reported that certain viruses manipulate the Wnt/β-catenin pathway; however, its role in Chikungunya virus (CHIKV) infection has not yet been investigated. Hence, the focus of the current study was to evaluate the importance of the Wnt/β-catenin pathway in CHIKV infection using *in vitro*, *in vivo*, and *ex vivo* models. The study revealed that CHIKV infection suppressed the active β-catenin protein associated with Wnt/β-catenin signaling, which, in turn, led to the reduction of cyclin-D1 and upregulation of glycogen synthase kinase-3β. Inhibition of β-catenin by an inhibitor (iCRT14) showed a drastic reduction in the viral RNA, protein, and new viral particle formation *in vitro*. Next, it was found that β-catenin is important in different phases of viral life cycle. Notably, the treatment of CHIKV-infected C57BL/6 mice with this drug reduced the disease score substantially with a 91.7% decrease in the viral load. Similar result was observed in human peripheral blood mononuclear cell-derived monocyte-macrophage populations and in physiologically relevant cells like RAW264.7 and C2C12 cells. Additionally, knockdown of β-catenin by siRNA drastically reduced viral particle formation. Further investigation revealed that CHIKV-nsP2 interacts with β-catenin during CHIKV infection. In conclusion, this work demonstrated for the first time the mechanistic insights regarding the role of the Wnt/β-catenin pathway for CHIKV infection that might contribute to the designing of effective therapeutics for the control of this virus infection in future.

**IMPORTANCE:**

Being obligate parasites, viruses use various host cell machineries in effectively replicating their genome, along with virus-encoded enzymes. In order to carry out infection and pathogenesis, viruses are known to manipulate fundamental cellular processes in cells and interfere with host gene expression. Several viruses interact with the cellular proteins involved in the Wnt/β-catenin pathway; however, reports regarding the involvement of protein components of the Wnt/β-catenin pathway in Chikungunya virus (CHIKV) infection are scarce. Additionally, there are currently no remedies or vaccines available for CHIKV. This is the first study to report that modulation of the Wnt/β-catenin pathway is crucial for effective CHIKV infection. These investigations deepen the understanding of the underlying mechanisms of CHIKV infection and offer new avenue for developing effective countermeasures to efficiently manage CHIKV infection.

## INTRODUCTION

Chikungunya virus (CHIKV) is a mosquito-borne virus and belongs to *Alphavirus* genus and *Togaviridae* family. It is transmitted by the *Aedes aegypti* and *Aedes albopictus* mosquitoes (1). The virus causes acute fever and joint pain, which can be severe and debilitating. Other symptoms may include headache, muscle pain, joint swelling, rash, and fatigue (2). CHIKV was first identified in Tanzania in 1952, and since then, outbreaks have occurred in many nations of Africa, Asia, Europe, and the Americas. CHIKV has a single-stranded positive-sense RNA genome that is approximately 11.8 kilobases in length and contains open reading frames that encode for structural and non-structural proteins (3). The rapid transmission of this virus and the unavailability of a specific medication or vaccine have made it a worldwide health problem. The virus is typically spread by mosquitoes, but it can also be carried from mother to child during childbirth (4). The incubation period of this virus ranges from 2 to 12 days, and the symptoms usually last for several days to weeks. There is no specific treatment for CHIKV, and the management of the disease is primarily focused on relieving the symptoms. This includes the use of nonsteroidal anti-inflammatory drugs to relieve fever and joint pain, as well as rest and hydration to reduce fatigue (1).

As soon as CHIKV enters the cell, it tries to modulate the host environment for efficient viral replication. By disrupting numerous signaling, cytoskeletal, and regulatory pathways that regulate crucial biological activities, CHIKV manipulates the host cell. CHIKV is known to interfere with the host replication process, disrupt the cell cycle (5), and alter the innate and adaptive immune responses of host cells (1). A deeper comprehension of CHIKV host cell interactions and the elucidation of the molecular mechanisms and cellular pathways hijacked by this virus are prerequisites for the development of novel antiviral approaches.

The canonical Wnt/β-catenin pathway is one significant signaling pathway that has been demonstrated to be modulated by viruses (6). β-Catenin is a key factor that governs the canonical Wnt/β-catenin signaling pathway. In the cytoplasm, β-catenin usually stays dormant; this is accomplished by its connection with a protein complex that includes adenomatous polyposis coli, glycogen synthase kinase-3β (GSK-3β), and casein kinase 1. Once bound, β-catenin is phosphorylated, and this complex continuously aides the proteasome-mediated destruction of β-catenin, assuring low levels of the β-catenin protein in the cell. A signaling cascade is activated when Wnt ligands bind to the heterodimeric Frizzled/low-density lipoprotein receptor-related protein-5/6 receptors present on the target cells. Breakdown of the complex initiates the accumulation of stable hypophosphorylated β-catenin in the cytosol, which is accompanied by its migration to the nucleus. Once it enters the nucleus, it attaches to DNA-binding proteins called T cell-specific factor (TCF) and lymphoid enhancer-binding factor-1 and stimulates the transcription of genes related to cell maintenance, proliferation, and survival (7).

Numerous studies demonstrate a connection between the Wnt/β-catenin signaling pathway and viruses. Viruses can either stimulate or inhibit the Wnt/β-catenin signaling pathway. For instance, human cytomegalovirus (HCMV) suppresses the transcriptional activity of this pathway by triggering the sequestration and destruction of endogenous β-catenin (8), while, to prevent the Wnt/β-catenin signaling, the negative regulatory factor (Nef) of human immunodeficiency virus (HIV) interacts with the β-catenin protein (9). Other reports suggest that silencing β-catenin increases HIV transcription (10), while inducing Wnt/β-catenin pathway with LiCl treatment reduces HIV proliferative activity in peripheral blood mononuclear cells (11). Contrarily, when the Wnt/β-catenin signaling is activated, influenza virus gene expression and virus production increase, whereas when it is inhibited, influenza virus production is constrained by a decrease in viral RNA synthesis (12). The Wnt/β-catenin signaling pathway is triggered by the hepatitis C virus (HCV) NS5A protein (13). Additionally, the PI3K/Akt pathway was activated by the LMP2A protein of Epstein-Barr virus, which also caused the deactivation of the GSK-3β protein. It also raised the amount of β-catenin in the cytoplasm and induced nuclear export of β-catenin (14).

Until date, the Wnt/β-catenin pathway has not been studied in CHIKV; an attempt was made to understand the significance of this essential pathway in CHIKV infection and to unveil the mechanism by which CHIKV exploits the Wnt/β-catenin pathway using *in vitro*, *in vivo*, and *ex vivo* models.

## MATERIALS AND METHODS

### Cells, virus, antibodies, and inhibitor

African green monkey kidney epithelial Vero, RAW 264.7 (mouse monocyte/macrophage), and human embryonic kidney 293T (HEK293T) cells were procured from the National Centre for Cell Science, India. The C2C12 (mouse myoblast) cell line was a generous gift from Dr. Amresh Panda, Institute of Life Sciences, India. The RAW 264.7 cells were cultured in Roswell Park Memorial Institute (RPMI) 1640 medium (Gibco RPMI 1640 GlutaMAX; Invitrogen, CA) supplemented with 10% fetal bovine serum (FBS) (Gibco FBS, Invitrogen, CA), gentamycin, and penicillin-streptomycin. The Vero, HEK293T, and C2C12 cells were maintained in Dulbecco’s modified Eagle’s medium (DMEM, PAN Biotech, Germany) supplemented with 10%–15% FBS (PAN Biotech, Germany), along with gentamycin and penicillin-streptomycin (Sigma, USA). All cells were grown at 37°C with 5% CO_2_. The Indian CHIKV strain (accession no. EF210157.2) was a kind gift from Dr. M.M. Parida (Defence Research Development Establishment, Gwalior, India). The CHIKV-nsP2 and nsP3 antibodies used in this study were developed by our group (15, 16). The CHIKV-E2 antibody was a generous gift from M.M. Parida (Defence Research Development Establishment, Gwalior, India). The total β-catenin, active β-catenin, and GSK-3β antibodies were procured from Cell Signaling Technologies (Cell Signaling, Inc., USA). The phospho-β-catenin antibody was a kind gift from Dr. Rupesh Dash, Institute of Life Sciences, India. The cyclin-D1 antibody (Santa Cruz) was a gift from Dr. Shantibhusan Senapati, Institute of Life Sciences, India. Glyceraldehyde 3-phosphate dehydrogenase (GAPDH) antibody was obtained from Abgenex, India. For Western blot, all primary antibodies were used at 1:1,000 dilution except nsP2 (1:3,000) and GAPDH (1:10,000). The iCRT14 inhibitor was acquired from Sigma Aldrich, USA. The MG132 inhibitor was a kind gift from Dr. Rupesh Dash, Institute of Life Sciences, India.

### Cytotoxicity assay

To assess the cytotoxicity of iCRT14 inhibitor in Vero, RAW264.7, and C2C12 cells, MTT (3-[4,5-dimethylthiazol-2-yl]-2,5 diphenyl tetrazolium bromide) assay was performed using the EZcount MTT cell assay kit (Himedia, India) as per the manufacturer’s protocol. One day before the experiment, nearly 20,000 cells were plated in a 96-well plate. After attaining 80% confluence, the cells were treated with several concentrations of the inhibitor for 15 h, and dimethyl sulfoxide (DMSO) was serving as the reagent control. The percentage of metabolically active cells was compared with the control cells, and the cellular cytotoxicity was estimated as described previously (17).

### Viral infection

The Vero, RAW264.7, HEK293T, and C2C12 cells were infected with different multiplicities of infection (MOIs) of CHIKV according to the protocol described before (5, 16). The cells and the supernatants were collected for several subsequent experiments.

### Plaque assay

To estimate viral titer, plaque assay was executed with the mock, infected, and infected with drug-treated cell supernatants. After infection, the cells were washed with 1× phosphate-buffered saline (PBS) and overlaid with DMEM containing methylcellulose (Sigma, USA) for 4 days at 37°C. The cells were fixed with 8% formaldehyde and stained with crystal violet, plaques were counted, and virus titers were exhibited as plaque-forming units per milliliter (PFU/mL) as described previously (5).

### Inhibitor treatment at different phases of viral infection

For most experiments, the Vero cells were infected with CHIKV (MOI, 0.1) and were incubated with DMSO or inhibitor post-treatment for 15 h post infection (hpi) except for RAW 264.7 and C2C12 cells (post-treatment were for 9 and 12 hpi).

For time-of-addition experiment, the Vero cells were infected with CHIKV for 90 minutes, and cells were washed with 1× PBS. The iCRT14 inhibitor (20 µM) was added into the fresh media at 0, 2, 4, 6, 8, 10, 12, and 14 hpi. DMSO was used as a control. The supernatants were collected at 15 hpi, and viral titers were estimated by plaque assay as described above (18).

In order to understand the role of β-catenin in different phases of viral infection, the β-catenin inhibitor (10 µM) was added at different times of infection (pre, during, post, and pre-during-post).

In pre-treatment condition, the Vero cells were pre-treated with iCRT14 inhibitor for 2 h. Cells were washed with 1× PBS followed by infection for 90 minutes. Next, the cells were washed, and fresh media without any inhibitor were added. Supernatant was collected at 15 hpi.

In case of treatment during the infection, the virus inoculum was mixed with the inhibitor and immediately used for infecting the cells for 90 minutes. Following this, cells were washed, and fresh media without any inhibitor were added. Supernatant was collected at 15 hpi.

For the post-treatment experiment, infected cells were washed with 1× PBS, and the inhibitor was added into the fresh media at 0 hpi, and supernatant was collected at 15 hpi.

For pre-during-post infection condition, the drug was present before, during, and after infection. Finally, supernatant was collected at 15 hpi.

All the supernatants were executed for plaque assay.

### Confocal microscopy

Immunofluorescence studies were executed as described earlier (17). Briefly, the Vero cells were grown on the coverslips; cells were infected with CHIKV and incubated with 20 µM of iCRT14 inhibitor for 8, 12, and 15 hpi. DMSO was used as a control. Furthermore, cells were washed with 1× PBS, fixed with 4% paraformaldehyde, and were incubated with primary antibodies of viral proteins E2 and nsP2 and host protein active β-catenin for 1 h followed by incubation with secondary antibodies of the Alexa Fluor 488 (anti-mouse) and Alexa Fluor 594 (anti-rabbit) (Invitrogen, MA) for 45 minutes. The cells were stained with 4,6-diamidino-2-phenylindole (DAPI; Life Technology) and mounted with antifade reagent (Invitrogen) to reduce photobleaching. The Leica TCS SP5 confocal microscope (Leica Microsystems, Germany) was used for acquiring images.

### Animal studies

Animal experiments were executed in strict compliance with the Committee for the Purpose of Control and Supervision of Experiments on Animals of India. All procedures and tests were reviewed and permitted by the Institutional Animal Ethics Committee (ILS/IAEC-274-AH/MAY-22). Mice aged 10 to 12 days were infected subcutaneously at the flank region of the hind limb with 10^7^ PFU of CHIKV, while control mice were administered serum-free media as previously described (5, 18). The mice in the treated group (*n* = 3) received oral doses of iCRT14 (6 mg/kg) at every 24-h interval up to 4 days post infection (dpi). Solvent was given to infection-control group (*n* = 3) of mice. Body weight was measured daily. For the clinical score studies, all mice (*n* = 6 mice in three groups) were observed regularly up to 5 dpi, and disease outcomes were noted as follows: no symptoms, 0; fur rise, 1; hunchback, 2; one hind limb paralysis, 3; both hind limb paralysis, 4; and death, 5. At 5 dpi, mice were sacrificed. The muscle tissue was used to determine viral load. The muscle and spleen tissues were snap frozen in liquid nitrogen for Western blot analysis.

### Reverse transcription-quantitative PCR (RT-qPCR)

Cells were harvested, and TRIzol (Invitrogen, USA) was used to extract the RNA. The obtained RNA was subjected to cDNA synthesis using the First Strand cDNA synthesis kit (Invitrogen, USA) with an equal volume of RNA. For RT-qPCR, CHIKV-specific primers for the E1 gene and host-specific primers for cyclin-D1 and GAPDH genes were used. Similarly, equal quantity of supernatants was used to extract viral RNA using the QIAamp viral RNA minikit (Qiagen, Germany) as per the manufacturer’s instructions. The RNA copy number was obtained with the Ct values plotted against the standard curve (19).

### siRNA transfection

Gene silencing of β-catenin was performed by using siRNA as previously described (5). Briefly, monolayers of HEK293T cells after reaching 70% confluency were transfected with Lipofectamine 2000 (Thermo Fisher Scientific, USA) using siRNA corresponding to β-catenin mRNA sequence (sense strand GUUGCUUGUUCGUGCACAUdTdT, GCUUAUGGCAACCAAGAAAdTdT, CAUGCAGUUGUAAACUUGA dTdT) or with scrambled siRNA as a negative control. Transfected cells were infected with CHIKV with MOI of 0.1. The supernatants and cells were collected after 15 hpi and assayed for plaque assay and Western blot analysis.

### Plasmid transfection

Overexpression of β-catenin was performed by using human β-catenin pcDNA3 plasmid according to the manufacturer’s protocol. Briefly, monolayers of HEK293T cells after reaching 70% confluency were transfected with Lipofectamine LTX (Thermo Fisher Scientific, USA) using human β-catenin pcDNA3 plasmid or with empty vector as a negative control. Transfected cells were infected with CHIKV with MOI of 0.1. The supernatants and cells were collected after 15 hpi and assayed for plaque assay and Western blot analysis.

### Western blot

Samples were lysed and analyzed by the Western blot as described (20). Briefly, the samples were harvested at different time points according to the experiments, and an equal volume of radioimmunoprecipitation assay (RIPA) buffer was used to lyse the cells. Snap-frozen mouse tissues were homogenized and lysed in RIPA buffer using the syringe lysis method. The proteins were separated on 10% SDS-polyacrylamide gel and transferred onto polyvinylidene difluoride membrane. The membrane was probed using antibodies as recommended by the manufacturer. The Immobilon Western chemiluminescent horseradish peroxidase substrate (Millipore, USA) was used to develop the blots. The quantification of protein bands from three independent experiments was conducted using the Image J software.

### Co-immunoprecipitation

Mock and CHIKV-infected Vero and C2C12 cells were harvested at 6 hpi. The cells were then lysed using the RIPA buffer and subjected to co-immunoprecipitation using the Dynabead protein A immunoprecipitation kit (Thermo Fisher Scientific, USA) as described previously (5, 20).

### Isolation of human peripheral blood mononuclear cells (hPBMCs), CHIKV infection, and iCRT14 treatment

hPBMCs were extracted from the blood collected from three healthy donors as previously described (18). Human blood was drawn from healthy donors following the guidelines of the Institutional Ethics Committee, NISER, Bhubaneswar. The reference number for the Institutional Ethics Committee (IEC)/Institutional Review Board is NISER/IEC/2022-04. Written informed consent was obtained from the participants’ legal guardian/next of kin. In brief, after 5 days, all the adherent cells were detached. The MTT assay was carried out using varied concentrations of iCRT14. The adherent cells were subjected to CHIKV infection at an MOI of 5 for 2 h and treated with 10 µM concentration of iCRT14. At 12 hpi, infected cells and supernatants were collected. The cells were fixed with 4% paraformaldehyde, followed by surface and intracellular staining for immunophenotyping of adherent population and detection of viral protein E2, respectively, using flow cytometry. For immunophenotyping, fluorochrome-conjugated anti-human CD90, CD11b, CD14, and CD19 antibodies (Abgenex, India) were used as previously described (18). Supernatants were used to determine viral titer by plaque assay.

### Statistical analysis

All the statistical analyses were performed using the GraphPad Prism version 8.0.1 software. Data are shown as mean ± SD for three independent experiments. For the comparison of two groups, unpaired two-tailed Student’s *t*-test was performed. For three or more groups, the one-way or two-way analysis of variance (ANOVA) with Bonferroni post-test was used. *P* values are specified at each figure or at their respective legend, and figure symbols represent *P < 0.05, **P < 0.01, ***P < 0.001, and ****P < 0.0001.

## RESULTS

### The Wnt/β-catenin pathway is dysregulated upon CHIKV infection

**Fig 1:**
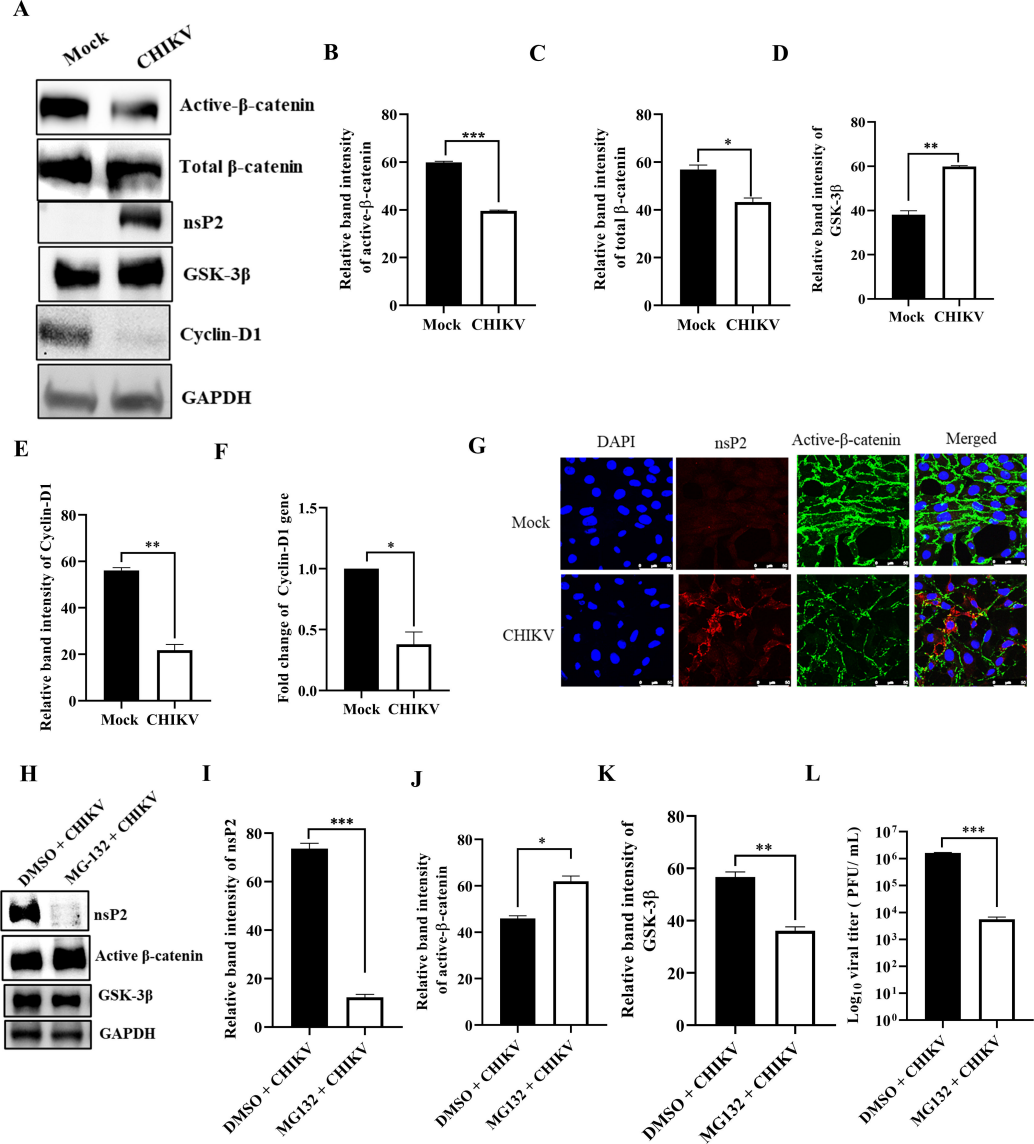
The Wnt/β-catenin pathway is dysregulated upon CHIKV infection. The Vero cells were mock or CHIKV infected and harvested at 6 hpi. (**A**) Western blot was performed using the nsP2, active β-catenin, total β-catenin, GSK-3β, cyclin-D1, and GAPDH antibodies. (**B–E**) Bar diagrams showing relative band intensities of active β-catenin, total β-catenin, GSK-3β, and cyclin-D1. Data of three independent experiments are shown as mean ± SD. (**F**) Total RNA was isolated from the mock and CHIKV-infected cells, and the cyclin-D1 gene was amplified by RT-qPCR. Bar diagram displaying the fold changes of cyclin-D1. (**G**) Mock or infected Vero cells were stained with the active β-catenin and nsP2 antibodies. Nuclei were counterstained with DAPI. Scale bar, 50 µm. (**H**) The Vero cells were CHIKV infected or treated with MG132 and harvested at 10 hpi. Western blot was performed using the nsP2, active β-catenin, GSK-3β, and GAPDH antibodies. (**I–K**) Bar diagrams showing relative band intensities of nsP2, active β-catenin, and GSK-3β. (**L**) Bar diagram depicting the log_10_ of viral titer of CHIKV + DMSO and CHIKV + MG132 supernatants obtained by the plaque assay. *n* = 3; *P ≤ 0.05, **P ≤ 0.01, and ***P ≤ 0.001 were considered statistically significant.

To assess whether the Wnt/β-catenin pathway is modulated in CHIKV infection, the levels of active β-catenin, total β-catenin, GSK-3β, and cyclin-D1 were investigated. Vero cells were infected with CHIKV at an MOI of 2 and harvested at 6 hpi. The protein levels were estimated by the Western blot. The protein levels of active β-catenin, total β-catenin, and cyclin-D1 were reduced significantly, whereas GSK-3β was upregulated following CHIKV infection compared to mock as shown in Fig. 1A through E. Next, to examine whether cyclin-D1 was transcriptionally activated by β-catenin, the level of cyclin-D1 mRNA following CHIKV infection was checked. Interestingly, the data demonstrated that there was 62% reduction in the level of cyclin-D1 after CHIKV infection (Fig. 1F). Moreover, immunofluorescence analysis was carried out to assess the active β-catenin level after CHIKV infection, and the results exhibited a decrease in active β-catenin level after CHIKV infection compared to mock (Fig. 1G). To further investigate whether loss of β-catenin expression after CHIKV infection is via normal proteasomal degradation, the Vero cells were treated with a known proteasomal inhibitor, MG132. The results revealed elevated level of active β-catenin and decreased level of GSK-3β after MG132 treatment compared to the DMSO control as shown in Fig. 1H, J, and K. Notably, nsP2 was also reduced by 83.3% compared to the DMSO control as shown in Fig. 1H and I. Moreover, in presence of MG132, there was 80% reduction in the viral titer compared to the DMSO control (Fig. 1L). The data suggest that inhibition of the proteosome rescues active β-catenin expression. Collectively, these results indicate that the Wnt/β-catenin pathway is impaired following CHIKV infection.

### The β-catenin protein of the Wnt/β-catenin pathway is crucial for CHIKV infection

**Fig 2:**
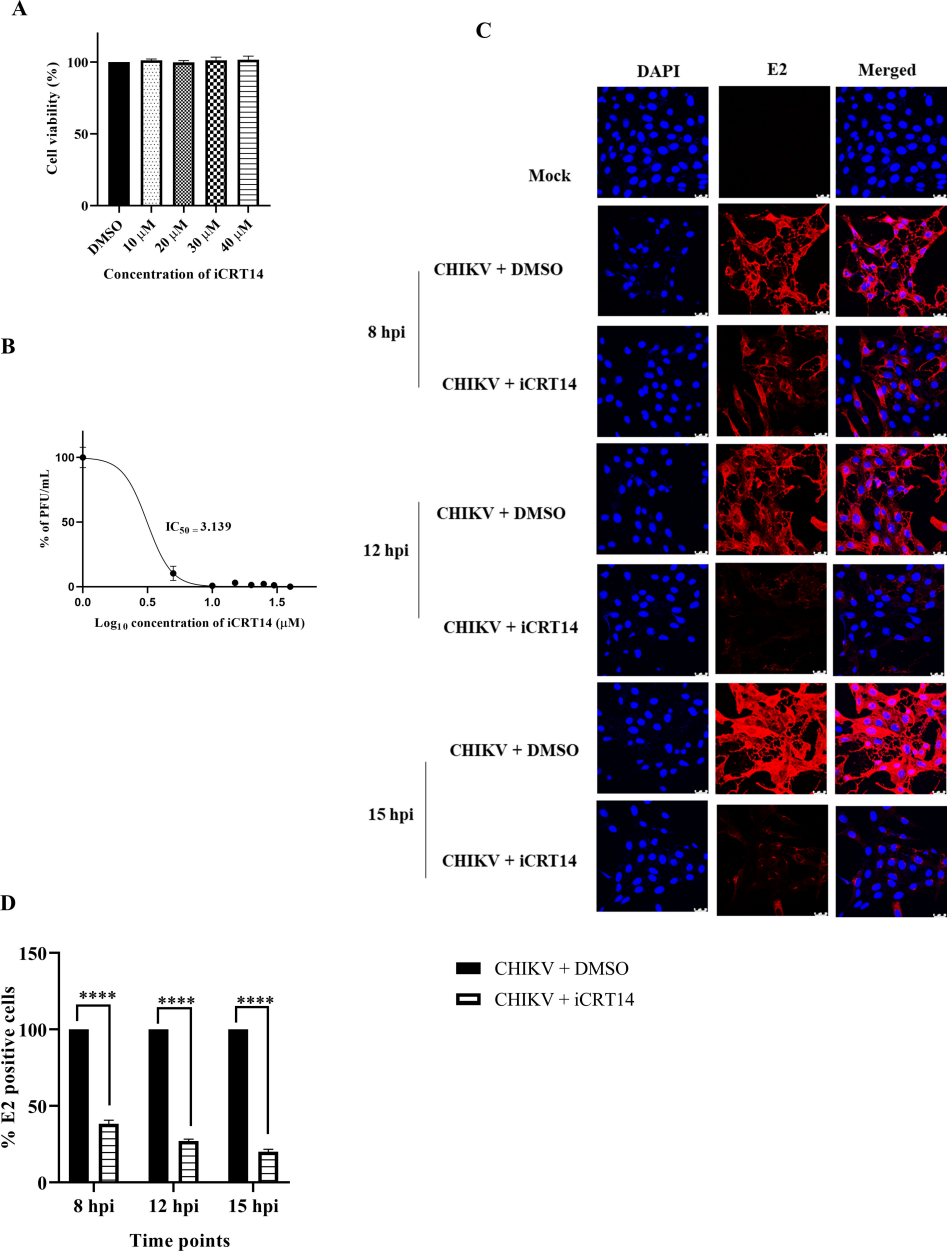
β-Catenin of the Wnt/β-catenin signaling is crucial for CHIKV infection. The Vero cells were treated with different concentrations (10, 20, 30, and 40 µM) of iCRT14 for 15 h, and the cytotoxicity of the cells was estimated by the MTT assay. (**A**) Bar diagram showing the viability of cells. (**B**) The IC_50_ of iCRT14 against CHIKV virus in Vero cells was estimated. The X-axis illustrates the logarithmic value of the different concentrations of iCRT14, and the Y-axis portrays the percentage of PFU/mL. (**C**) Immunofluorescence analysis exhibiting mock, infected, and iCRT14-treated Vero cells stained with E2 antibody at 8, 12, and 15 hpi. Nuclei were counterstained with DAPI. Scale bar, 10 µm. (**D**) Bar diagram representing the percent positive E2 cell counts from the confocal images. Data of three independent experiments are shown as mean ± SD. ****P ≤ 0.0001 was considered statistically significant.

To comprehend the significance of the Wnt/β-catenin signaling pathway in CHIKV infection, a specialized inhibitor of β-catenin (iCRT14) was used. The MTT assay was executed to assess the cytotoxicity of the inhibitor in Vero cells. Cells were found to be viable at all the concentrations (10, 20, 30, and 40 µM) of the drug used as shown in Fig. 2A. The IC_50_ of iCRT14 was found to be 3.1 µM (Fig. 2B). The anti-CHIKV potency of iCRT14 was further demonstrated using confocal microscopy, which revealed a substantial decrease in the viral E2 protein of CHIKV compared to respective DMSO control at 8, 12, and 15 hpi (Fig. 2C and D). Hence, these findings indicate that the β-catenin of the Wnt/β-catenin signaling pathway is important for CHIKV infection.

### The iCRT14 inhibitor lowers viral RNA, protein, and viral load

**Fig 3:**
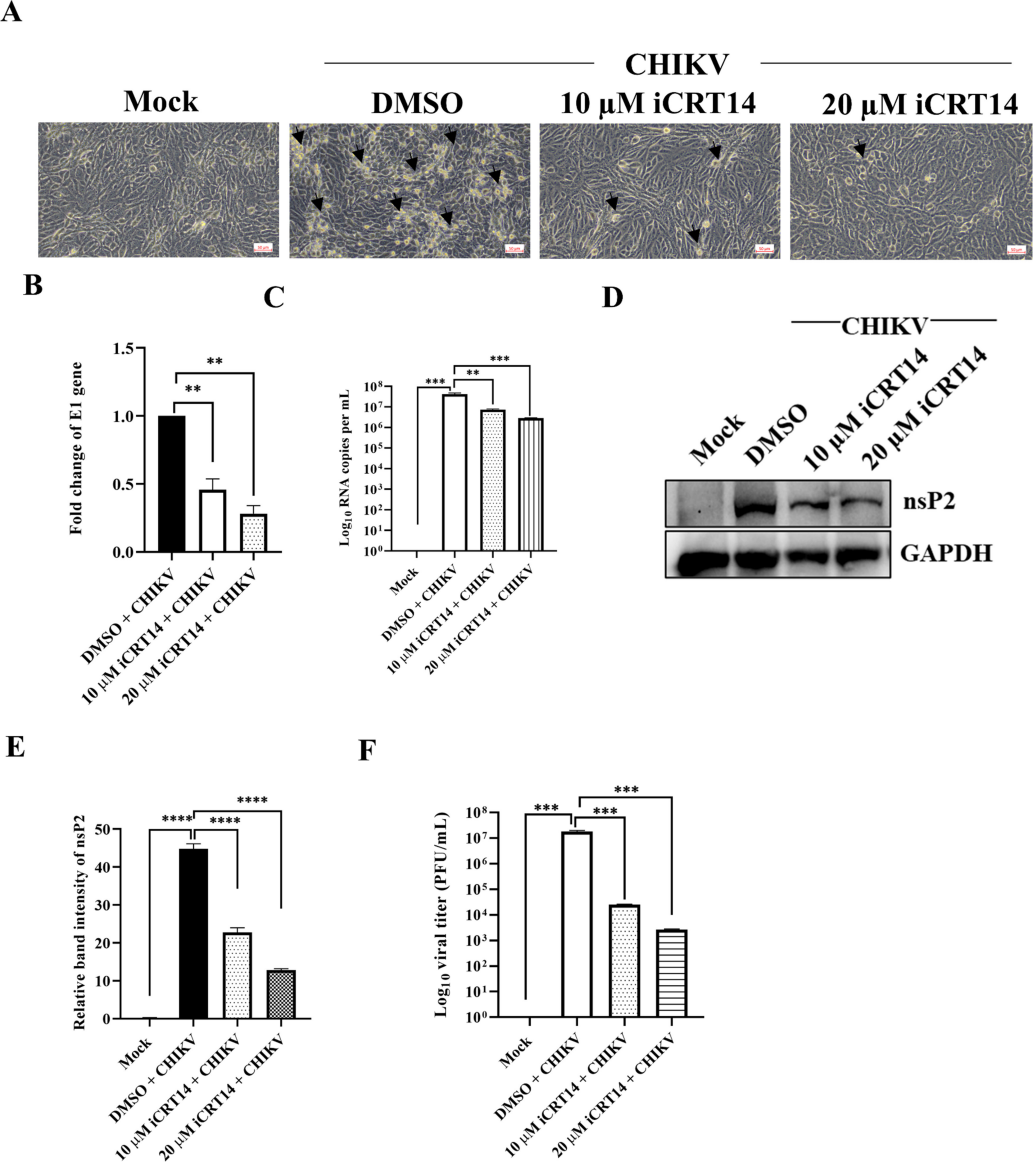
The iCRT14 inhibitor lowers viral RNA, protein, and viral load. The Vero cells were treated with different concentrations (10 and 20 µM) of iCRT14 for 15 h. (**A**) Image depicting the CPE of cells, which was observed under a microscope (magnification −20×) with the different concentrations of drug. (**B**) Total RNA was isolated from the mock, CHIKV-infected, and drug-treated cells, and the CHIKV-E1 gene was amplified by RT-qPCR. Bar diagram displaying the fold changes of viral E1 gene. (**C**) Bar diagram showing CHIKV RNA copy number per milliliter in virus-infected and drug-treated culture supernatants. (**D**) Western blot image showing nsP2 level of mock, infected, and infected plus drug-treated cells. GAPDH served as a loading control. (**E**) Bar diagrams depicting the relative band intensity of nsP2. (**F**) Bar diagrams depicting the log_10_ of viral titer obtained by the plaque assay. Data of three independent experiments are shown as mean ± SD. **P ≤ 0.01 and ***P ≤ 0.001 were considered statistically significant.

To determine the effect of iCRT14 on virus infection, cytopathic effect (CPE) was observed under a microscope followed by CHIKV infection and drug treatment (10 and 20 µM). Comparing the drug-treated cells to the DMSO control, a considerable decrease in CPE was observed (Fig. 3A). Next, the E1 gene was amplified from total RNA and viral RNA, respectively. The data demonstrated that the level of E1 gene expression had decreased by 55% and 72% after 10 and 20 µM drug treatment, respectively (Fig. 3B). Additionally, the effect of 10 and 20 µM concentrations of iCRT14 was checked on CHIKV viral copy numbers from virus-infected and iCRT14-treated cell culture supernatant. The RT-qPCR data revealed 81.9% and 93.1% reduction in the CHIKV viral copy numbers in the culture supernatants as shown in Fig. 3C. Notably, Western blot was carried out, which showed a 49.2% and 71.4% considerable and dose-dependent decrease in nsP2 level using 10 and 20 µM drug, respectively (Fig. 3D and E). Additionally, this was verified by plaque assay, where drastic reduction was observed in the viral particle formation after drug treatment compared with the DMSO control (Fig. 3F). As a result, it is possible to infer that iCRT14 can reduce CHIKV infection by lowering viral RNA, protein, and viral load.

### β-Catenin is essential in the early stage of the CHIKV life cycle

**Fig 4:**
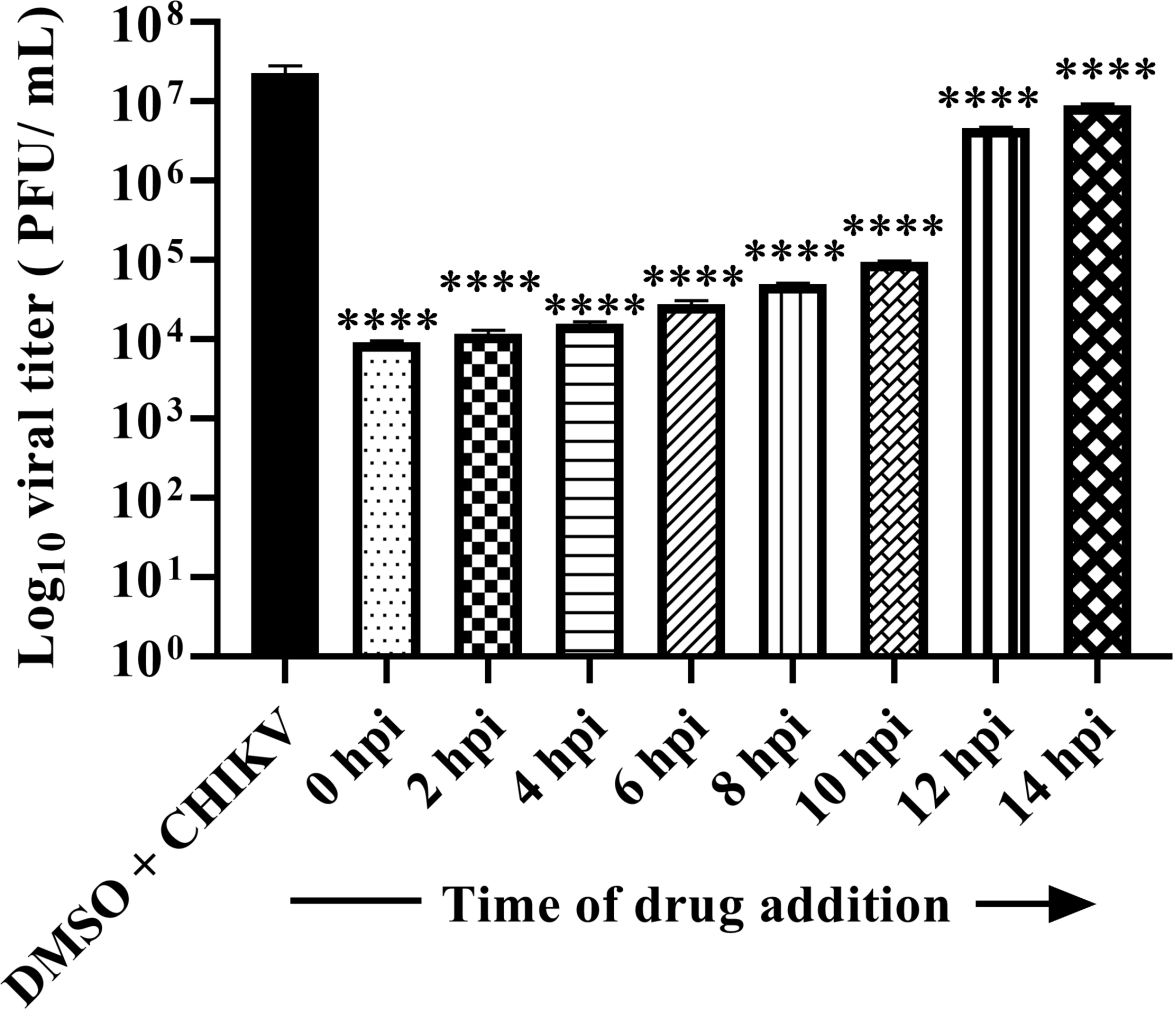
β-Catenin is essential in the early stage of the CHIKV life cycle. Vero cells were infected by CHIKV at an MOI of 0.1, and 20 µM of iCRT14 was added to each sample every 2 h up to 14 hpi. The bar diagram represents the log_10_ of viral titer for all the samples from the supernatants that were collected at 15 hpi, which were obtained by plaque assay. Data of three independent experiments are shown as mean ± SD. ****P ≤ 0.0001 was considered statistically significant.

A “time-of-addition” experiment was conducted to elucidate at which stage(s) of the CHIKV life cycle iCRT14 exhibits its antiviral effect. The inhibitor exerted maximum inhibition in the early stage (0 to 6 hpi) of viral replication (>95%) in progeny virus released into the cell culture medium compared to the DMSO control. In contrast, it had reduced antiviral effect (up to 60% reduction in viral titer) when added between 8 and 14 hpi (Fig. 4). The outcome demonstrates that β-catenin is important in the early stage of the CHIKV life cycle.

### The iCRT14 inhibitor shows anti-CHIKV activity at different phases of viral infection

**Fig 5:**
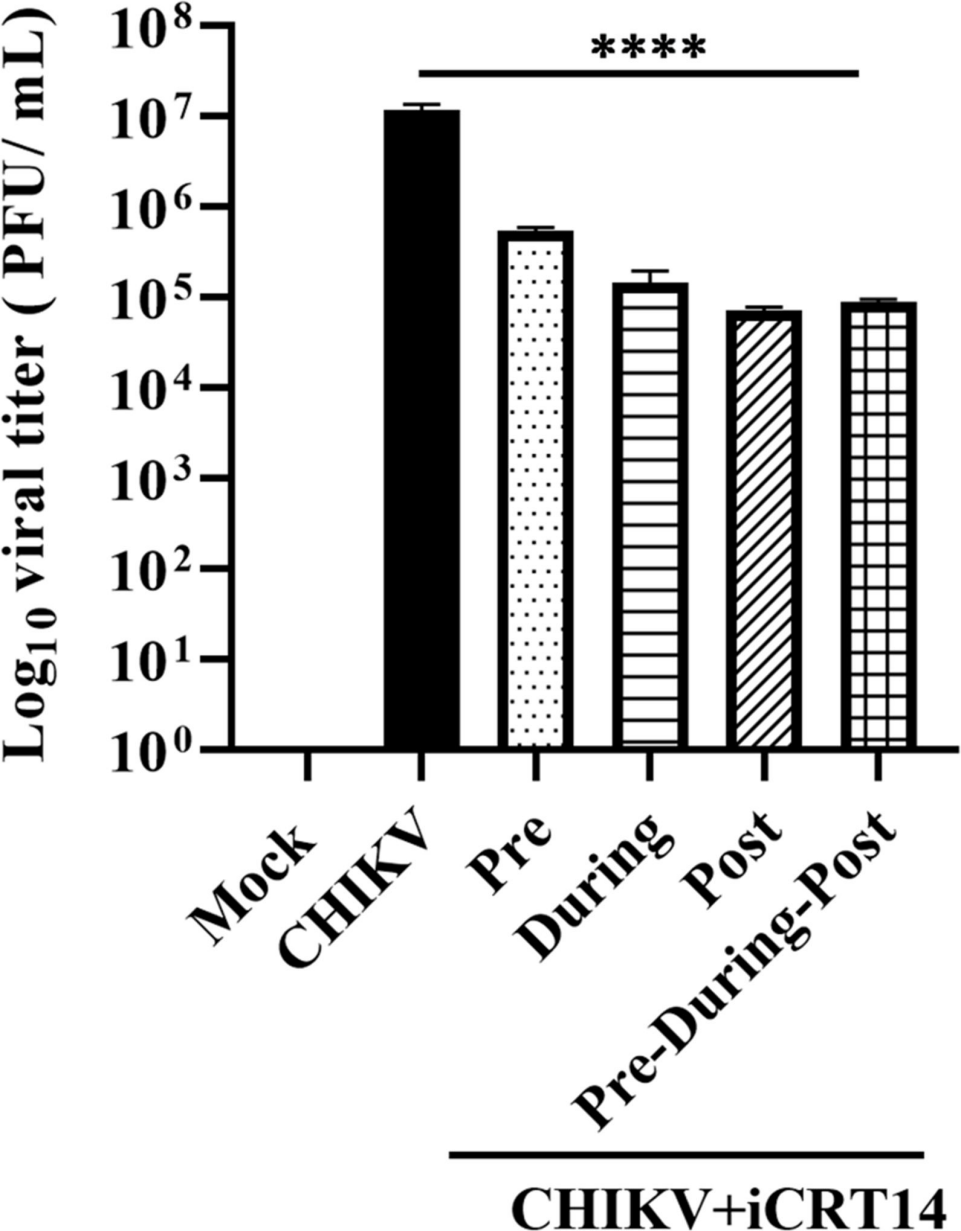
The iCRT14 inhibitor shows anti-CHIKV activity at different phases of viral infection. Vero cells were treated with iCRT14 (10 µM) separately before (2 h prior to infection), during (90 minutes during infection), and post infection (for 15 h). The drug was also present before, during, and after infection for pre-during-post infection condition. Supernatants were collected at 15 hpi cells and subjected for plaque assay. The bar diagram shows the log_10_ of viral titer. Data presented as mean ± SD. *n* = 3; ****P ≤ 0.0001 was considered statistically significant.

The iCRT14 inhibitor was administered at various stages of CHIKV infection in order to assess the involvement of β-catenin in pre-treatment, during treatment, and post-treatment along with another condition where the inhibitor was present in all the three above-mentioned conditions. iCRT14 showed anti-CHIKV activity in all the conditions. The findings revealed that pre-, during, and post treatment with iCRT14 reduced viral progeny formation by 94%, 94.5%, and 95.8%, respectively, while the combined (pre-during-post) treatment led to 95.1% decrease in viral titer compared with the DMSO control (Fig. 5). Together, it can be suggested that β-catenin might be required in different phases of CHIV life cycle for efficient infection.

### Efficient inhibition of CHIKV infection by iCRT14 in mice

**Fig 6:**
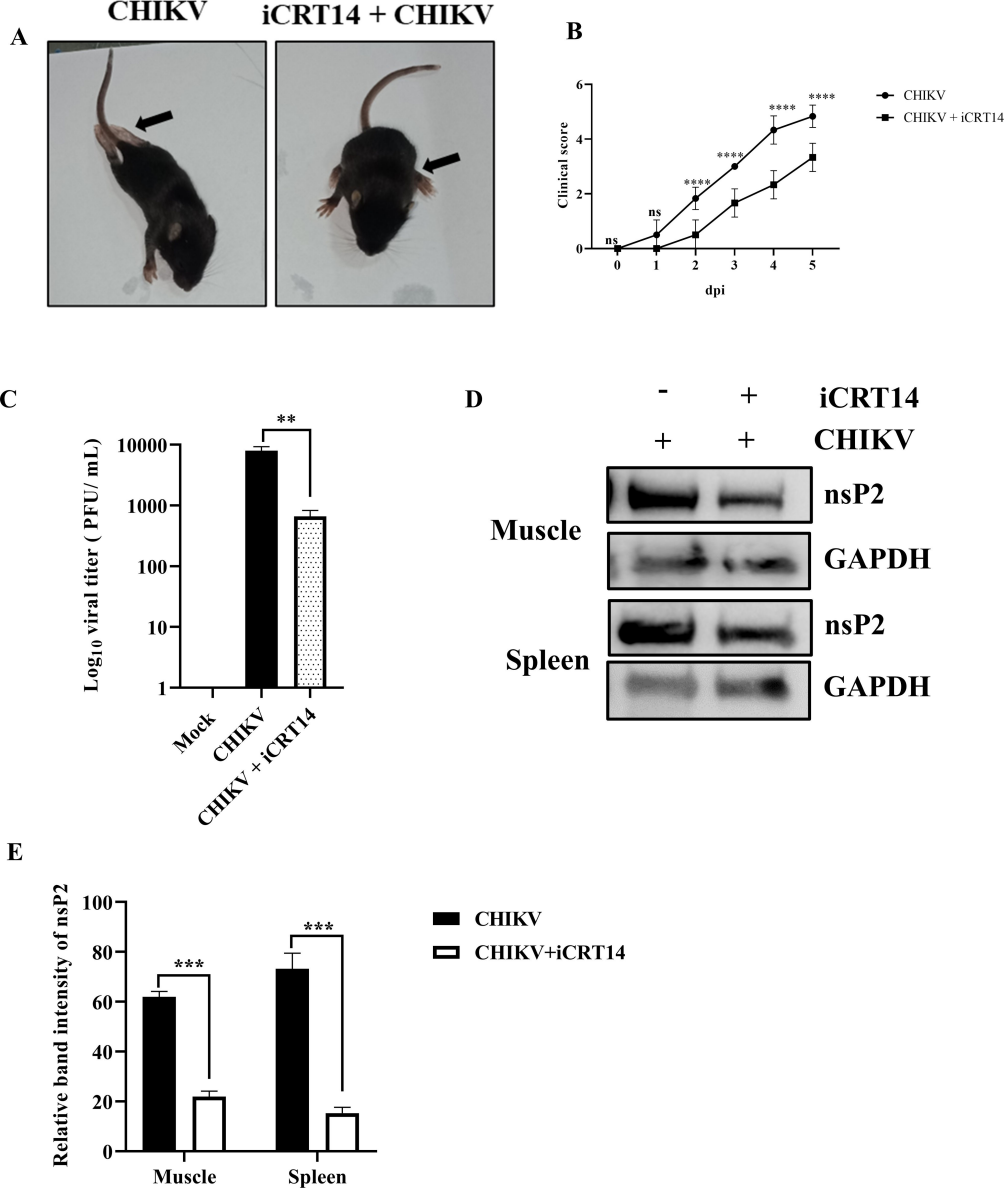
Efficient inhibition of CHIKV infection by iCRT14 in mice. C57BL/6 mice were infected subcutaneously with 10^7^ PFU of CHIKV and treated with 6 mg/kg of iCRT14 at 24-h intervals up to 4 dpi. Mice were sacrificed at 5 dpi; muscle and spleen tissues were collected for further experiments. (**A**) Images of CHIKV-infected and drug-treated mice at 4 dpi, the arrow point showing the development of hind limb paralysis in infected mice. (**B**) For the clinical score studies, disease outcomes were noted as follows: no symptoms, 0; fur rise, 1; hunchback, 2; one hind limb paralysis, 3; both hind limb paralysis, 4; and death, 5. Graph representing the clinical scores of the disease symptoms of mice during CHIKV infection which were monitored from 1 dpi to 5 dpi (*n* = 6). (**C**) Bar diagram showing the log_10_ of viral titer of virus-infected and drug-treated mice muscle tissue samples. (**D**) Western blot showing the viral nsP2 protein in muscle and spleen tissue samples. GAPDH was used as loading control. (**E**) Bar diagram exhibiting the relative band intensities of nsP2 in muscle and spleen tissue samples from infected or drug-treated mice, and it was obtained through the GraphPad Prism software (*n* = 3) where the two-way ANOVA with Sidak’s post-test was used. Data of three independent experiments are shown as mean ± SD. **P ≤ 0.01, ***P ≤ 0.001, and ****P ≤ 0.0001 were considered statistically significant.

The efficacy of iCRT14 for its antiviral potential was tested using 10- to 12-day-old C57BL/6 mice. Mice were infected with 10^7^ particles of CHIKV subcutaneously. They were given 6 mg/kg iCRT14 at 24-h intervals for 5 dpi. The treated mice showed a lower clinical score compared to the untreated mice, which displayed gradual hind limb paralysis (Fig. 6A) and mortality (Fig. 6B). Plaque assay results demonstrated that the viral load in the iCRT14-treated mice was reduced by 91.7% compared with the control group, as determined using muscle tissue samples from various groups (Fig. 6C). In addition, Western blot analysis showed that iCRT14 treatment reduced nsP2 protein levels by 64.6% and 79.2% in the muscle and spleen, respectively (Fig. 6D and E). These results indicate that iCRT14 can effectively reduce CHIKV infection in mice.

### Optimal level of β-catenin is essential for efficient CHIKV infection

**Fig 7:**
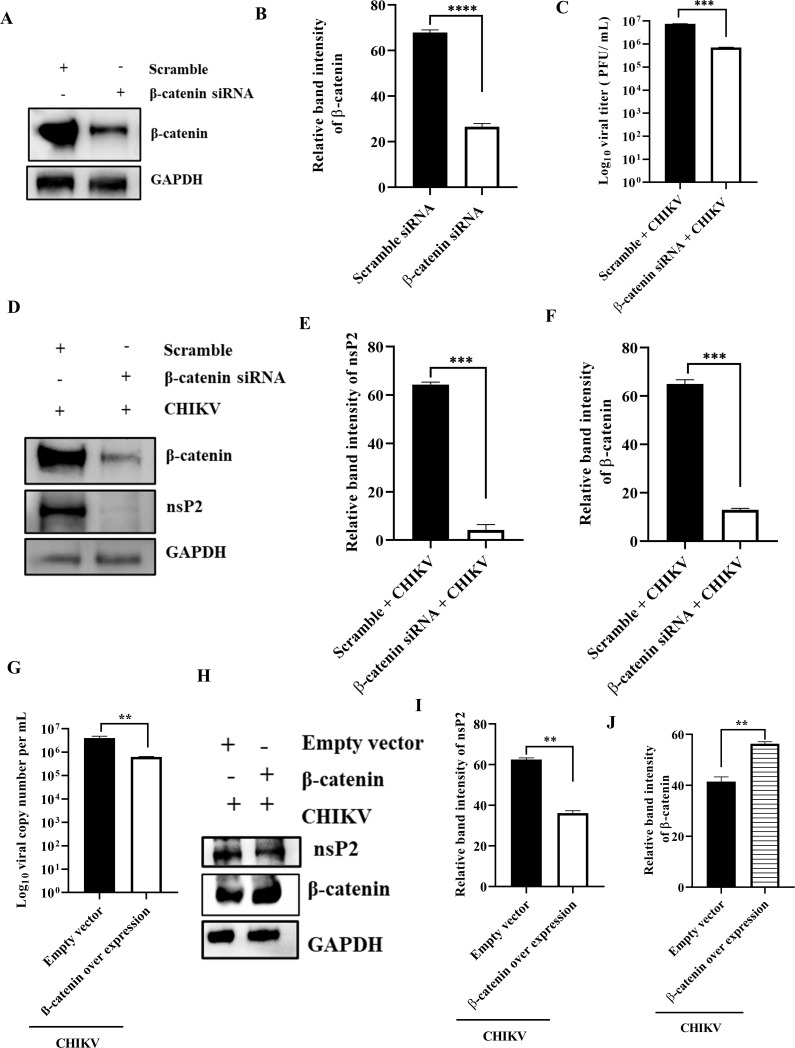
Optimal level of β-catenin is essential for efficient CHIKV infection. The HEK293T cells were transfected with scramble siRNA or 30 pm of β-catenin siRNA. (**A**) β-Catenin level was estimated by Western blot, and GAPDH was used as a loading control. (**B**) Bar diagram showing relative band intensity of the β-catenin protein. After 24 hpt, cells were infected with CHIKV (MOI, 0.1) and harvested at 15 hpi for further downstream experiments. (**C**) Bar diagram representing the log_10_ of viral titer in the cell supernatant of scramble + CHIKV and β-catenin siRNA + CHIKV samples, which were obtained by plaque assay. (**D**) Western blot exhibiting the nsP2 and β-catenin protein levels after transfection and infection with CHIKV. (**E** and **F**) Bar diagrams depicting relative band intensities of the nsP2 and β-catenin proteins. β-Catenin was overexpressed in HEK293T cells using the β-catenin plasmid. At 24 hpt, cells were infected with CHIKV (MOI, 0.1) and harvested at 15 hpi for further downstream experiments. (**G**) Bar diagram representing the log_10_ of viral titer in the cell supernatant of empty vector + CHIKV and β-catenin overexpression + CHIKV samples, which were obtained by plaque assay. (**H**) Western blot analysis depicting nsP2 and β-catenin protein levels after transfection and infection with CHIKV. (**I and J**) Bar diagrams depicting relative band intensities of the nsP2 and β-catenin proteins. Data of three independent experiments are shown as mean ± SD. **P ≤ 0.01, ***P ≤ 0.001, and ****P ≤ 0.0001 were considered statistically significant.

In order to examine the significance of the β-catenin protein for CHIKV infection, β-catenin was silenced in HEK293T cells using 30-pm siRNA. At 24 hours post transfection (hpt), cells were collected and processed for the Western blot to determine the levels of β-catenin. As compared to the scramble siRNA control, the β-catenin protein level was found to have decreased by 60.8%. (Fig. 7A and B). Then, CHIKV (MOI, 0.1) was used to infect the siRNA-transfected cells. After 15 hpi, the supernatants and cell lysates were gathered and processed for plaque assay and Western blot, respectively. Notably, 90% less viral particles were produced compared with the scramble siRNA control (Fig. 7C). Like this, Western blot analysis revealed 93.5% reduction in nsP2 protein levels following siRNA transfection (Fig. 7D and E). β-Catenin level was also diminished significantly (Fig. 7D and F). Moreover, β-catenin was overexpressed in HEK293T cells using the β-catenin plasmid. At 24 hpt, CHIKV (MOI, 0.1) was used to infect the cells. After 15 hpi, the supernatants and cell lysates were collected and processed for plaque assay and Western blot, respectively. Notably, 84.4% less viral particles were produced compared with the empty vector control (Fig. 7G). Like this, Western blot analysis revealed 42.3% reduction in nsP2 protein levels following β-catenin overexpression (Fig. 7H and I). β-Catenin level was elevated significantly (Fig. 7H and J). These findings collectively imply that optimal level of β-catenin is essential for efficient CHIKV infection.

### CHIKV-nsP2 interacts with β-catenin during CHIKV infection

**Fig 8:**
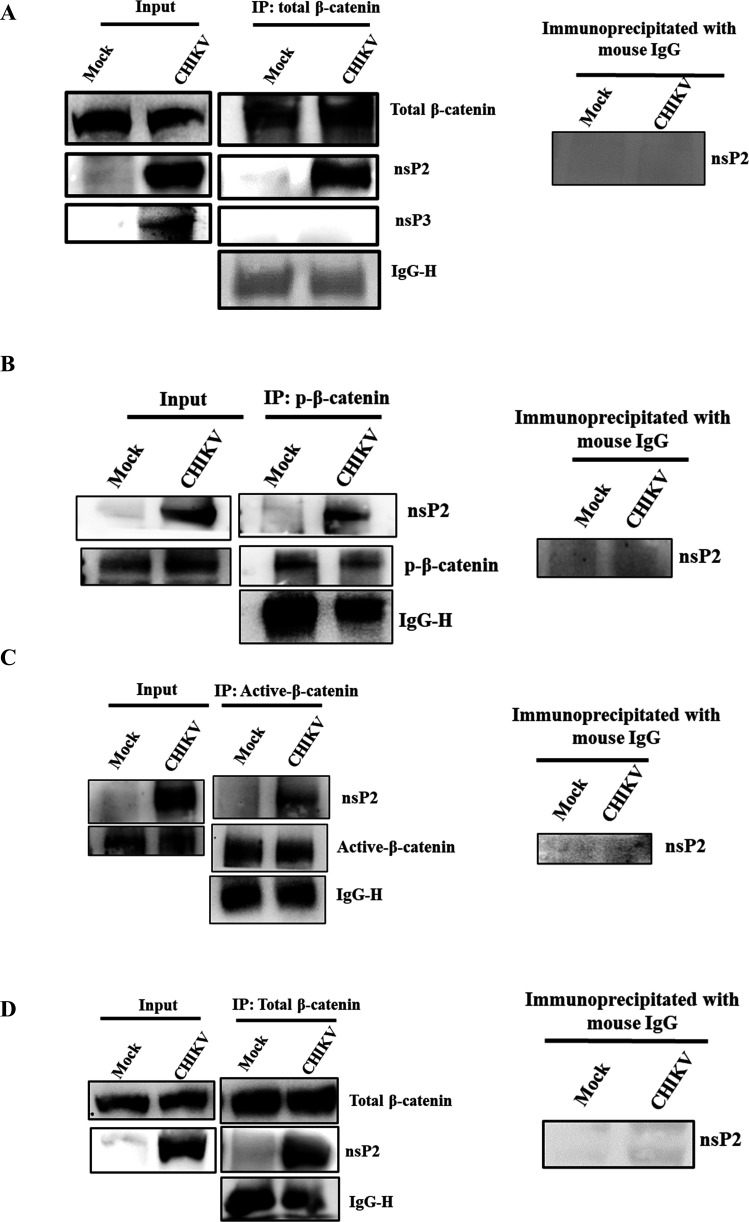
CHIKV-nsP2 interacts with the host β-catenin protein during CHIKV infection. The Vero and C2C12 cells were infected with CHIKV and harvested at 6 hpi. The cell lysates were co-immunoprecipitated with the total β-catenin, phospho-β-catenin, active β-catenin, and nsP3 antibodies. (**A**) Western blot analysis in Vero cells depicting the levels of total β-catenin, nsP2, and nsP3 in the whole cell lysate (left) and co-immunoprecipitation analysis showing the interaction of the CHIKV-nsP2 and β-catenin proteins (middle). Right panel represents the negative control, where normal mouse IgG was used to immunoprecipitate the protein complex and probed with the nsP2 antibody. (**B**) Western blot analysis in Vero cells depicting the levels of phospho-β-catenin and nsP2 in the whole cell lysate (left) and co-immunoprecipitation analysis showing the interaction of the CHIKV-nsP2 and phospho-β-catenin proteins (middle). Right panel represents the negative control, where normal mouse IgG was used to immunoprecipitate the protein complex and probed with the nsP2 antibody. (**C**) Western blot analysis in Vero cells depicting the levels of active β-catenin and nsP2 in the whole cell lysate (left) and co-immunoprecipitation analysis showing the interaction of the CHIKV-nsP2 and β-catenin proteins (middle). Right panel represents the negative control, where normal mouse IgG was used to immunoprecipitate the protein complex and probed with the nsP2 antibody. (**D**) Western blot analysis in C2C12 cells depicting the levels of total β-catenin and nsP2 in the whole cell lysate (left) and co-immunoprecipitation analysis showing the interaction of the CHIKV-nsP2 and total β-catenin proteins (middle). Right panel represents the negative control, where normal mouse IgG was used to immunoprecipitate the protein complex and probed with the nsP2 antibody.

To understand if there is any interaction of β-catenin with viral protein, cells were infected with the virus. At 6 hpi, CHIKV-infected Vero cells were collected and subjected to co-immunoprecipitation and Western blot analysis. It was found that total β-catenin immunoprecipitated the CHIKV-nsP2 protein in the Vero cells. However, nsP3 does not interact with β-catenin. Here, IgG was considered as a negative control (Fig. 8A). Next, to investigate whether both phosphorylated and non-phosphorylated (active) forms of β-catenin precipitated by nsP2, co-immunoprecipitation experiments were performed with both phosphorylated and non-phosphorylated (active) β-catenin. It was found that nsP2 was precipitated by both the forms (Fig. 8B and C). Moreover, it was also found that total β-catenin was immunoprecipitated by the CHIKV-nsP2 protein in the C2C12 cells (Fig. 8D). These findings indicate that CHIKV-nsP2 interacts with β-catenin during CHIKV infection.

### The iCRT14 inhibitor diminishes CHIKV infection in the hPBMC-derived monocyte-macrophage populations

**Fig 9:**
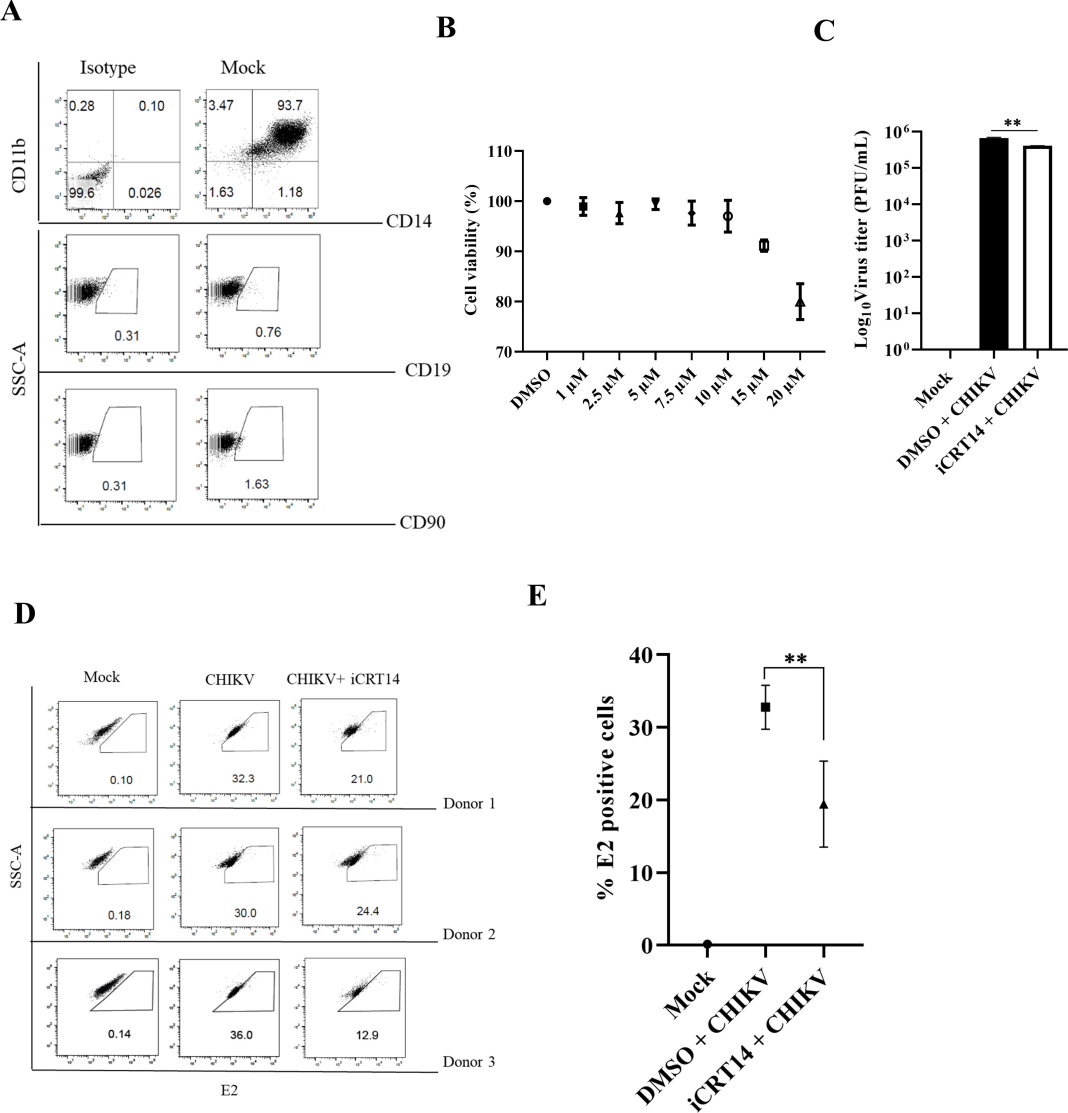
iCRT14 reduces CHIKV infection in the hPBMC-derived adherent cell populations. The human PBMCs were isolated from blood samples of three healthy donors and infected with CHIKV. (**A**) Dot plot showing the percentages of B cells (CD19), T cells (CD90), and CD14^+^CD11b^+^ monocyte-macrophage cells from adherent hPBMCs by flow cytometry. (**B**) The graph representing the cytotoxicity of iCRT14 in the hPBMC-derived adherent cell populations by the MTT assay. (**C**) Bar diagram depicting the log_10_ of viral titer obtained by plaque assay. (**D**) Dot plot showing the percentage of the viral E2-positive hPBMC-derived monocyte-macrophage population in mock, CHIKV-infected, and iCRT14-treated CHIKV-infected samples using flow cytometry. (**E**) The graph showing the percentage of positive cells for the CHIKV-E2 protein, as derived by flow cytometry assay. Data of three independent experiments are shown as mean ± SD. **P ≤ 0.01 was considered statistically significant.

It was intriguing to investigate whether iCRT14 can decrease CHIKV infection in monocyte-macrophage cells produced from hPBMC (CD14/CD11b1 cell populations). In order to characterize the hPBMC-derived adherent cell populations, specific markers for B cells (CD19), T cells (CD90), and monocyte-macrophage cells (CD11b and CD14) were stained using antibodies, followed by flow cytometry analysis (Fig. 9A). The data revealed that the adherent population was significantly predominant in CD14 + CD11b + monocyte-macrophage cells. After that, MTT assay was performed with different concentrations of iCRT14 (1, 2.5, 5, 7.5, 10, 15, and 20 µM). Furthermore, studies were carried out with 10 µM iCRT14 because 95% of cells remained viable in the presence of this compound (Fig. 9B). Interestingly, 40% less viral particles were produced in iCRT14-treated cells compared with the DMSO control (Fig. 9C). Next, the efficacy of iCRT14 was evaluated after CHIKV infection in the hPBMC-derived adherent populations of three healthy donors *ex vivo*. The E2-positive cell population in the CHIKV-infected populations was 32.76%, but treatment with 10 µM iCRT14 resulted in a decrease in infection to 19.4% (Fig. 9D and E). Thus, the data indicate that iCRT14 can reduce CHIKV infection in hPBMC-derived monocyte-macrophage populations *ex vivo*.

### The iCRT14 inhibitor impedes CHIKV infection in the physiologically relevant cells

**Fig 10:**
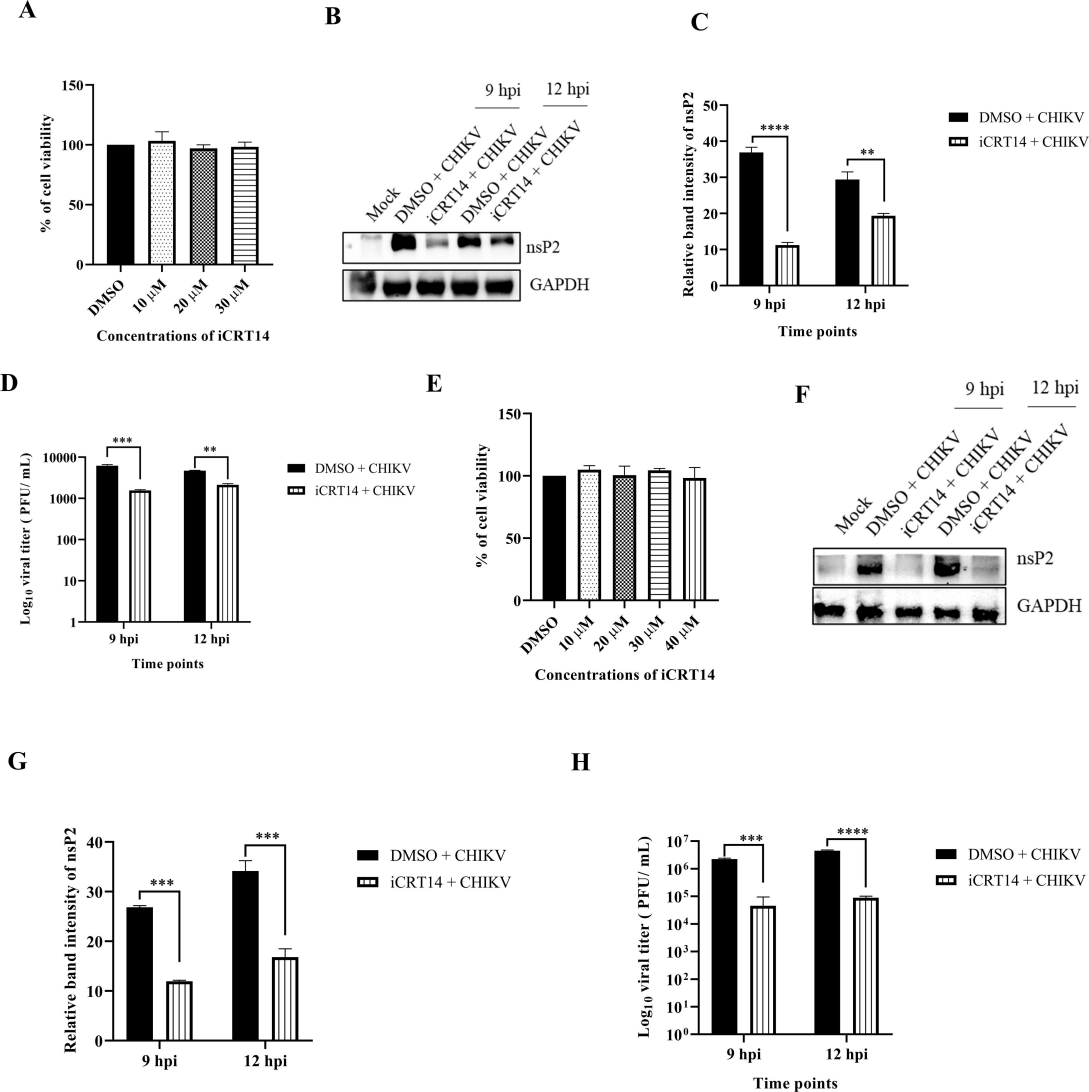
The iCRT14 inhibitor impedes CHIKV infection in the physiologically relevant cells. The RAW264.7 and C2C12 cells were treated with different concentrations (10, 20, 30, and 40 µM) of iCRT14, and the cytotoxicity of the cells was estimated by the MTT assay. (**A** and **E**) Bar diagrams showing the viability of cells in the RAW264.7 and C2C12 cells. The RAW264.7 and C2C12 cells were infected with CHIKV and treated with 20 µM iCRT14. The supernatants and cells were harvested at 9 and 12 hpi for both the cell lines and executed for Western blot and plaque assay. (**B** and **F**) Western blot images depicting the nsP2 protein level in mock, infected, and infected plus treated cells in RAW264.7 and C2C12 cells, respectively. GAPDH served as a loading control. (**C** and **G**) Bar diagrams representing the relative band intensity of nsP2 in RAW264.7 and C2C12 cells, respectively. (**D** and **H**) Bar diagrams depicting the log_10_ of viral titer obtained by plaque assay in RAW264.7 and C2C12 cells, respectively. Data of three independent experiments are shown as mean ± SD. *P* ≤ 0.05 was considered statistically significant. **P ≤ 0.01, ***P ≤ 0.001, and ****P ≤ 0.0001 were considered statistically significant.

Majority of the experiments in this study are performed in Vero cells that are highly permissive to CHIKV; therefore, RAW264.7 and C2C12 cells were used to evaluate the role of the Wnt/β-catenin pathway in CHIKV infection in physiologically relevant cells. To assess cytotoxicity of the inhibitor in RAW264.7 and C2C12 cells, the MTT assay was performed. Both cells were found to be viable at all used drug concentrations (10, 20, 30, and 40 µM) as shown in Fig. 10A and E. The cells were then infected with CHIKV and treated with 20 µM iCRT14. Cells and supernatants were harvested at 9 and 12 hpi for RAW264.7 and C2C12 cells, followed by Western blot and plaque assay. The data revealed that there was significant reduction in the nsP2 protein level following iCRT14 treatment in RAW264.7 (Fig. 10B and C) and C2C12 (Fig. 10F and G) cells. Notably, the virus titers were decreased by nearly 74.8% and 54% following iCRT14 treatment at 9 and 12 hpi in RAW264.7 cells, respectively (Fig. 10D). Moreover, 79% and 85% less viral particles were produced following iCRT14 treatment at 9 and 12 hpi in the C2C12 cells (Fig. 10H). Taken together, the data illustrate that the Wnt/β-catenin pathway is necessary for effective CHIKV infection in the RAW264.7 and C2C12 cells.

## DISCUSSION

For this crippling disease of CHIKV, there are currently no vaccines or treatments available. Potential therapies may be established with the help of an understanding of the interactions between the virus and the host pathways. In this study, the significance of the Wnt/β-catenin signaling pathway in CHIKV infection was investigated. To verify the importance of the β-catenin protein in CHIKV infection, iCRT14, a specific inhibitor of the Wnt/β-catenin signaling pathway, was used. The IC_50_ of iCRT14 against CHIKV was found to be 3.139 µM. Moreover, inhibition of the proteosome rescues active β-catenin expression. The anti-CHIKV potency of iCRT14 was further demonstrated using confocal microscopy, which revealed a substantial and dose-dependent decrease in the viral E2 protein of CHIKV. The iCRT14 inhibitor reduced CHIKV infection *in vitro* by lowering viral RNA, protein, and viral load, and β-catenin was found to be important in all the stages of viral infection. There was drastic reduction in the viral load in the iCRT14-treated CHIKV-infected C57BL/6 mice and hPBMC-derived monocyte-macrophages. Knockdown of β-catenin using siRNA reduced viral progeny formation by 90%, confirming that β-catenin is vital host factor for CHIKV infection. Furthermore, it was demonstrated that CHIKV-nsP2 interacts with β-catenin during CHIKV infection in the Vero and C2C12 cells.

In the current investigation, the Wnt/β-catenin signaling pathway was found to be downregulated upon CHIKV infection, which was comparable with the previous reports related to other viruses such as HCMV and HIV (8, 9). Furthermore, β-catenin was found to be translocated into the nucleus to stimulate the transcription of target genes such as cyclin-D1 (21). However, in the current study, it was observed that the level and translocation of the β-catenin protein to the nucleus were inhibited in CHIKV-infected cells. Additionally, the level of cyclin-D1, a protein involved in cell cycle regulation, was reduced, whereas the level of GSK-3β was augmented upon CHIKV infection compared to mock. These findings align with previous study where HCMV infection reduces the expression of β-catenin target genes and the Wnt-induced transcriptional activity of β-catenin in dermal fibroblasts and human placental extravillous trophoblasts (8). Next, in presence of MG132, there was drastic reduction in the viral titer and in the level of nsP2 compared to the DMSO control. It was also observed that the level of active β-catenin was elevated, while the level of GSK-3β was decreased compared to DMSO control. The results corroborated with previous report where treatment with the proteasome inhibitors MG132 and lactacystin leads to a decrease of viral titers in CHIKV-infected GripTite 293 MSR cells (22). Moreover, earlier studies reported that MG132 treatment reduced herpes simplex virus 1 (HSV-1) infection along with elevation of Wnt/β-catenin signaling pathway (23, 24). The current findings from the *in vitro* investigations demonstrated that the absence of β-catenin substantially decreased the viral titer and protein level of CHIKV in the Vero, RAW264.7, and C2C12 cells. Prior investigations have also emphasized similar results in case of certain viruses, such as the influenza virus, HSV-1, and SARS-CoV-2 where iCRT14 treatment reduced viral infections (12, 17, 25) . On the contrary, inhibition of β-catenin by iCRT14 increased the expression of certain viral proteins in bovine herpesvirus type 1 (26).

The *in vitro* results have been strengthened by using the C57BL/6 mouse model and *ex vivo* experiments. The infected mice exhibited severe illness with gradual weight loss, tiredness, slumped posture, arthritis, and immobility, while iCRT14 treatment (6 mg/kg) alleviated these symptoms by lowering the viral titer and protein. These outcomes are consistent with previous report where mice were partially protected against influenza virus infection by the intraperitoneal dose of iCRT14. This also alleviated clinical symptoms and lowered viral load (12). Further studies shed light on the possible impact of iCRT14 at various stages in the CHIKV life cycle. Interestingly, it turned out that iCRT14 might potentially impede with early phase of the viral life cycle. It also drastically reduced the viral titer when β-catenin was reduced in the pre-treatment and during treatment conditions. However, there was less impact on virus replication when inhibitor was added after the 6 hpi. These results indicate the potential of iCRT14 to target several stages of the CHIKV life cycle along with the host pathway to regulate infection. Earlier report exhibited that 2-h pre-treatment was essential for the antiviral effect of iCRT14 in HSV-1 (25). Knockdown of β-catenin using β-catenin-specific siRNA led to 90% reduction in the viral load, suggesting the important role of β-catenin for CHIKV infection, which aligns with previous reports. Blocking of the Wnt/β-catenin signaling pathway by silencing of β-catenin reduced avian leukosis virus subgroup J infection in chicken embryo fibroblast (CEF) cells and SARS-CoV-2 infection in Vero cells (17, 27).

Moreover, it was demonstrated that CHIKV-nsP2 interacts with β-catenin during CHIKV infection in the Vero and C2C12 cells. Reports have shown that the Nef protein of HIV interacts with the host β-catenin protein. A region of Nef comprises of amino acids that resemble the β-catenin binding regions on the native β-catenin ligand. This was further validated *in vitro* and in a co-immunoprecipitation experiment using HEK293 cells. Additionally, it was demonstrated that the NS5A protein of HCV, alone or in conjunction with p85, has the capability to directly interact with the host β-catenin protein. Notably, both the N and C termini of NS5A were found to contribute to this interaction (9, 28).

**Fig 11:**
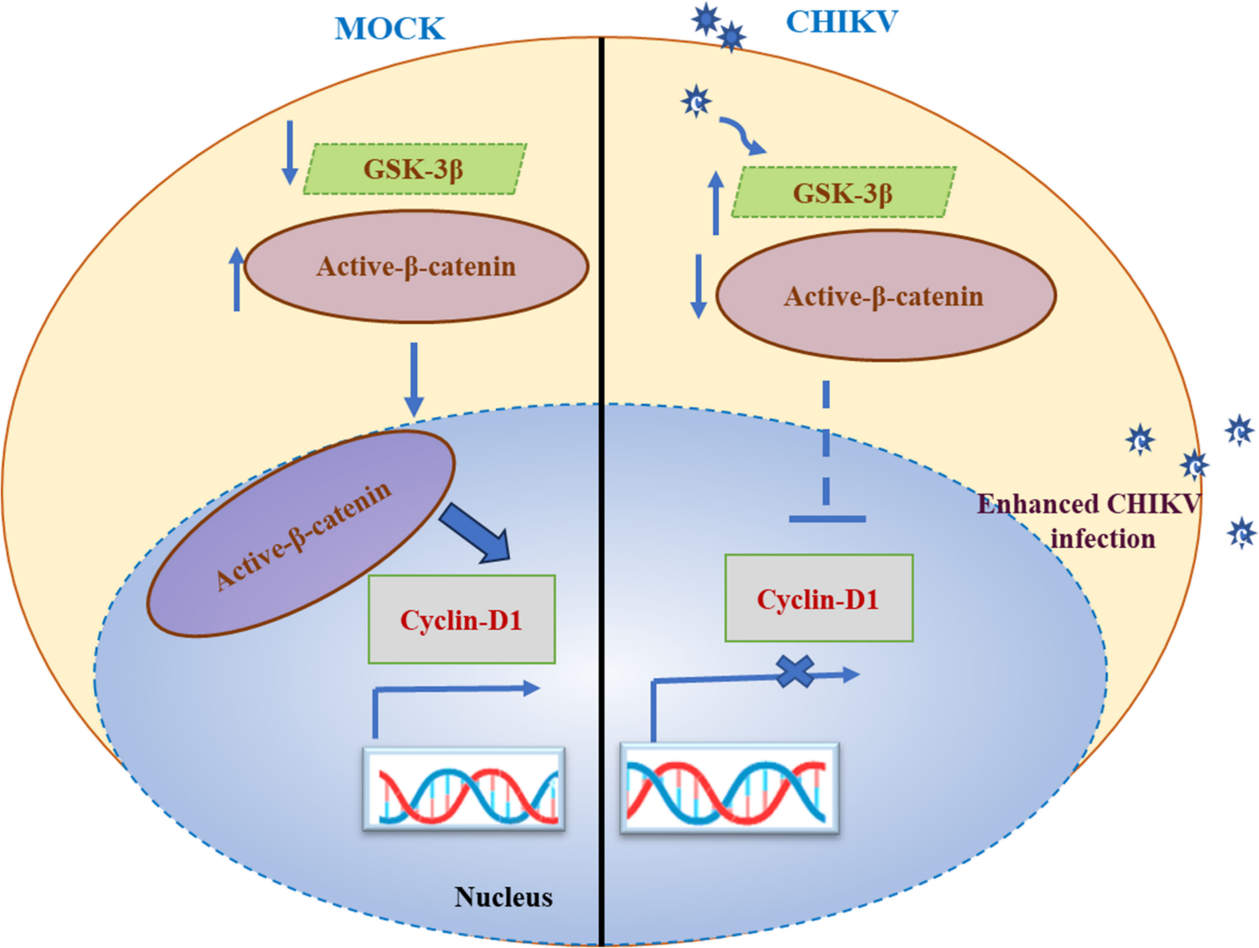
Working model portraying the dysregulation of the Wnt/β-catenin signaling by the CHIKV infection. In the current investigation, it was found that CHIKV infection led to upregulation of GSK-3β and reduction in the level of active β-catenin. This subsequently reduces the level of cyclin-D1 for efficient viral production.

For the virus to exert dominance over the host cell, it must be able to interfere with apoptosis and cytoskeletal rearrangement, and this control would probably be required at multiple stages post infection. The vastness and intricacy of the Wnt/β-catenin pathway make a straightforward response inconceivable, but parameters including the stage of viral infection and cell type are apparent choices. In case of HIV, suppressing either β-catenin or TCF-4 induces HIV long terminal repeat activity. While the Wnt/β-catenin signaling was inhibited, the level of HIV replication was enhanced. However, it should be noted that certain studies have indicated human primary astrocytes subjected to HIV showed increased level of Wnt2B and Wnt10B. The Wnt5a mRNA expression was increased in mouse spinal dorsal horn neuronal cells after HIV infection (10, 11, 29, 30). It is more likely that CHIKV has the potential to modulate the function of this critical pathway at different phases throughout the viral life cycle. Moreover, targeting the components of the Wnt pathway by inhibitors in human also has some side effects. The blockade of Wnt signaling causes side effects such as impairment of tissue homeostasis and regeneration and the normal Wnt-dependent stem cell population may be affected (31). Earlier report suggests that excessive doses of tankyrase inhibitor can result in gastrointestinal toxicity, which may limit the clinical use of similar inhibitors (32). Additionally, the Wnt pathway controls a variety of components of bone development, and using Wnt inhibitors may have harmful effects on bone marrow (33). The use of small-molecule inhibitors to target the WNT signaling system in the clinical trial is still in its initial stages. Despite widespread investigation into the pathway, it is still unknown which strategy will offer both clinical efficacy and safety. In this study, the Wnt/β-catenin signaling pathway was investigated for the first time in CHIKV infection. This infection led to upregulation of GSK-3β level and reduction in the active β-catenin protein level, which subsequently reduce the level of cyclin-D1. This, in turn, led to efficient viral production (Fig. 11). To gain a better understanding, future investigations will be required to identify the specific amino acids involved in the interactions between β-catenin and nsP2. Furthermore, mutational studies could enhance the knowledge regarding the significance of this interaction in the context of CHIKV infection. Moreover, investigating the role of other players of the Wnt/β-catenin pathway in CHIKV infection and the interconnectedness of the Wnt/β-catenin pathway with other pathways can enhance the comprehension of viral pathogenesis. Therefore, the current study highlights the importance of the Wnt/β-catenin signaling pathway in CHIKV infection and suggests that this pathway may be a potential target for designing antiviral therapy against CHIKV infection in future.

## Data Availability

The data that support the findings of this study are available from the corresponding author upon reasonable request. Some data may not be made available because of privacy or ethical restrictions.
